# Implementing Targeted Sampling: Lessons Learned from Recruiting Female Sex Workers in Baltimore, MD

**DOI:** 10.1007/s11524-018-0292-0

**Published:** 2018-07-31

**Authors:** Sean T. Allen, Katherine H. A. Footer, Noya Galai, Ju Nyeong Park, Bradley Silberzahn, Susan G. Sherman

**Affiliations:** 1 Department of Health, Behavior and Society, Bloomberg School of Public Health, Johns Hopkins University, 624 N. Broadway, Baltimore, MD 21205 USA; 2 Department of Epidemiology, Bloomberg School of Public Health, Johns Hopkins University, 615 N. Wolfe St, Baltimore, MD 21205 USA

**Keywords:** Female sex workers, Targeted sampling, HIV prevention

## Abstract

Globally, HIV prevention interventions have proven efficacious among street-based female sex workers (FSWs); yet, there is a dearth of US-based HIV prevention research among this group. The lack of research among FSWs in the USA is partially driven by challenges in recruiting members of this population. The purpose of this research is to describe how targeted sampling was employed to recruit a cohort of street-based FSWs for a study that examined the role of police in shaping the HIV risk environments of street-based FSWs in Baltimore, MD. Our research demonstrates that targeted sampling can be an advantageous strategy for recruiting hidden populations that are mobile and geographically dispersed.

## Introduction

Female sex workers (FSWs) experience high rates of HIV and STI infections [1–3]. Socio-structural vulnerabilities (e.g., poverty, stigma, violence), the illicit nature of sex work, high rates of substance use, and other comorbid conditions place FSWs at sustained high risk of HIV acquisition [4–7]. Street-based FSWs are at heightened risk for HIV acquisition given their engaging in sex work in the context of extremely unsafe work environments [8–12]. Globally, HIV prevention interventions have proven efficacious among FSWs [13–17]; yet, there is a dearth of US-based HIV prevention research among this group. The lack of research among FSWs in the USA is partially driven by challenges in recruiting members of this population. The illegality and associated stigma of sex work in the USA may drive FSWs to be reluctant to engage in research and discuss the contexts of their sex work. Existing HIV studies have primarily employed two methods for recruiting “hidden” high-risk populations, targeted sampling (TS) and, more recently, respondent-driven sampling (RDS) [18–20].

TS was developed as a strategy to sample people who inject drugs (PWIDs) and their sexual partners during the early years of the HIV epidemic [18]. As noted in Watters (1989), TS is “an ongoing and interactive process in which data are constantly analyzed and used to adjust the recruitment and sampling techniques.” [18] This method involves analyses of secondary data sources as well as conducting primary data collection to identify areas where the target population may be found and, within those areas, locations that may be included in a sampling frame [18]. The flexible and on-going nature of TS allows investigators to incorporate new knowledge and revise recruitment strategies to better reflect the fluid nature and behaviors of the target population [18]. TS can be especially useful for the recruitment of street-based FSWs as although areas of concentrated sex trade are often identifiable within urban areas, the times and locations of sex work activity may fluctuate due to external factors such as police presence and weather conditions. Despite the strengths of TS, it has fallen in popularity, possibly due to the labor-intensive nature of its methods and the development of RDS, which can be used to reduce biases associated with non-random recruitment [21].

The changing landscape of publicly accessible datasets could fundamentally reduce the labor-intensive nature of TS. Increasing numbers of secondary datasets are available via online portals. These online data sources may have transformative impacts on how TS can be employed by reducing the amount of work required to implement the strategy, particularly in its initial phases and by allowing easy updates as new data becomes available. Secondary data can be analyzed using geospatial software to efficiently identify the locations where members of the target population congregate. Because TS fell out of favor in advance of this era of easily accessible secondary data sources via online platforms, there is a dearth of literature describing how it can leverage these datasets to inform participant recruitment. We aimed to describe how a diversity of secondary data sources was paired with primary ethnographic data to recruit a sample of FSWs via TS for a study that examined the role of police in shaping the HIV risk environments of street-based FSWs in Baltimore, MD. FSW recruitment described here was in advance of the Sex worker and Police Promoting Health in Risky Environments (SAPPHIRE) study.

## Methodology

### Overview of TS Approach Implementation

**Fig. 1:**
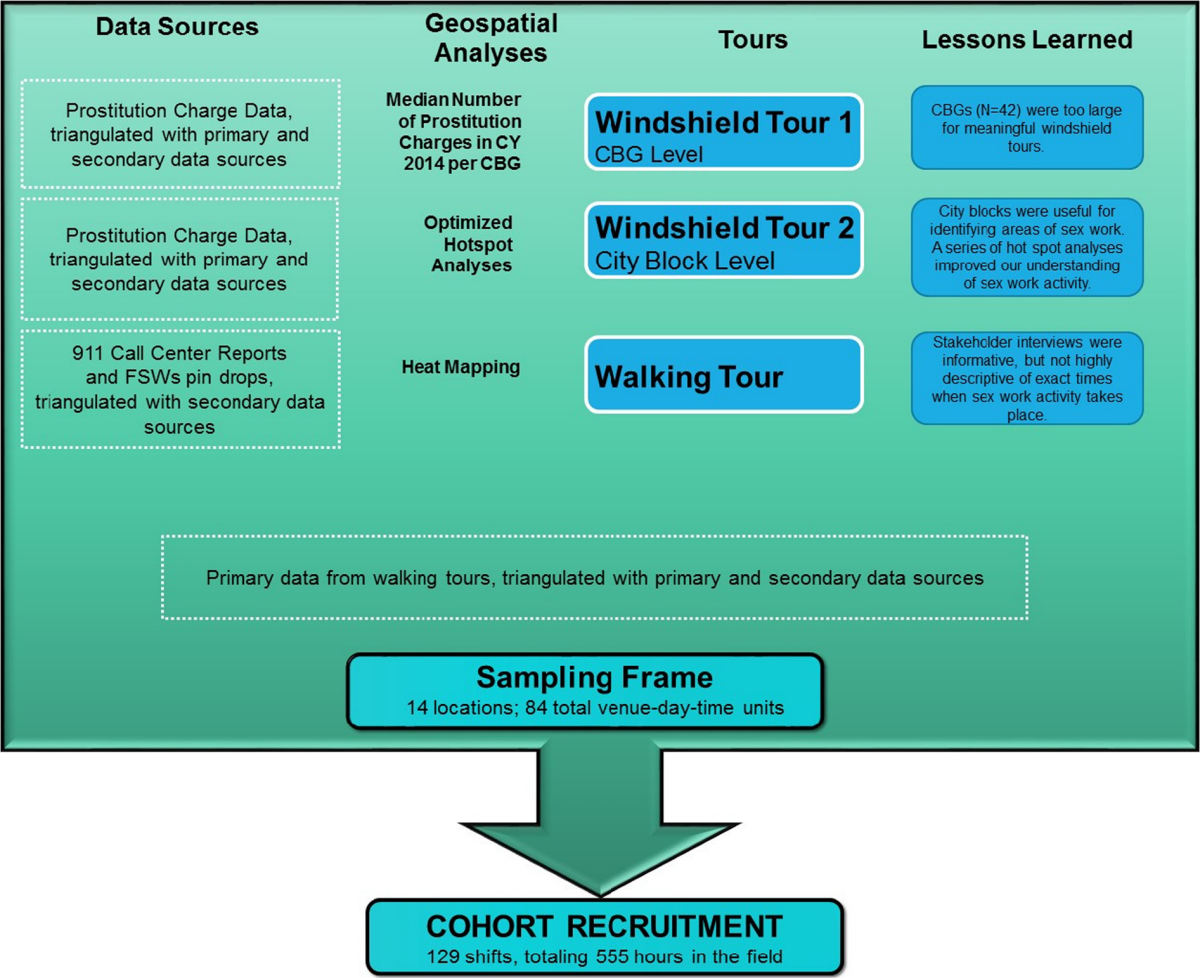
Overview of targeted sampling approach

In accordance with the tenets of TS, we first conducted thorough explorations of secondary data sources (e.g., prostitution charge data) that may include indicators of sex work activity. Given the hidden nature of sex work, we also engaged in primary data collection with local stakeholders (e.g., law enforcement) to understand the geotemporal distribution of sex work activities throughout Baltimore City, MD. We briefly describe our primary and secondary data sources below. We then provide an overview of our initial geospatial analyses to identify locations of sex work activity and create an exhaustive sampling frame. Figure 1 provides an overview of our TS approach.

#### Secondary Data Sources

There were three sources of secondary data for our TS strategy, all of which were publicly accessible online. First, we utilized data from an open access dataset [22] that described the top charge of persons processed at Baltimore’s Central Booking and Intake Facility, including information pertaining to the following: time and location of charge, charge descriptions, and basic demographics of the individual charged. Given data availability, only data from calendar year 2014 were included in the development of our TS strategy. Due to variations in how the charge descriptions were reported, we imported the data to SAS Enterprise Guide v4.3 and compressed all charge descriptions (i.e., removed all non-alphanumeric data). We then calculated frequencies for the compressed charge descriptions and determined specific text strings that could be used to identify prostitution charges. All records that did not suggest prostitution charges were deleted. Remaining data were inspected manually to validate that they were prostitution charges. In total, we identified 599 prostitution charges; however, only 75.6% (*n* = 453) reflected charges among persons who were identified as female and that had latitude and longitude coordinates of where the charge occurred.

Second, we used a publicly accessible dataset of 911 call center reports in Baltimore City from March through December 2015 [23]. Specifically, we extracted reports involving prostitution activity (*n* = 2193). Like the charge data, these reports included information pertaining to when and where the reported illicit activity took place. Although a complete calendar year of 911 call center data was not available at the time of analyses, we felt that the recency of the data enhanced our ability to identify locations of potential sex work.

Third, we extracted data from a publicly accessible “Johns website” in which persons created posts about their encounters with sex workers. The posts were heterogenous in the amount of information persons disclosed; for example, some posts included the following: costs per sex act; times and locations of sex work activity; adverse experiences with sex workers; drug use; and police activity. Notably, the locations of sex work activity were presented in multiple ways; for example, some postings included descriptions of street intersections where FSWs may be found while others detailed information pertaining to FSW strolls. There was also a post that included a map of approximate locations of sex work activity. Staff explored the website for information detailing FSW activities, but focused data extraction efforts on those postings that were created in the 2 months preceding participant recruitment. These data were then imported to ArcMap v10.2.1 for analysis.

#### Primary Data Sources

Primary data describing areas of sex work activity were derived from a parallel ethnographic study with the Baltimore City Police Department, a formative phase of the SAPPHIRE study (Footer et al., An ethnographic exploration of police practices towards street-based female sex workers in Baltimore City, USA – informing structural interventions to address HIV risk. under review). Study staff participated in 300 hours of ride-alongs with police officers in city districts with high numbers of prostitution charges, offering the opportunity to collect extensive geotemporal data of SW activity. During ride-alongs, ethnographers recorded all visual sightings of potential sex workers via dropping pins on Google Maps. Identification of potential sex workers was based on both law enforcement accounts (e.g., historical accountings of their interactions with known sex workers) and staff members’ judgment of the individual’s behavior, such as loitering in known sex work strolls or approaching vehicles. Similar data were also collected during windshield tours in which field staff drove around areas of potential sex work to confirm the presence of sex work activity and identify any potential safety concerns (described below). Windshield tours were also used in this research as they are a proven strategy to observe social behaviors in urban areas [24]. Ethnographic data of sex work activity were derived from a diverse group of stakeholders, including police leadership, patrol officers, and syringe services program staff. Lastly, primary data were derived from interviews with FSWs that served on a community advisory board for the SAPPHIRE study.

#### Geospatial Analyses and Windshield Tours

Maps that described the geographic divisions of Baltimore City were downloaded from the US Census Bureau and imported to ArcMap. These files were used to contextualize our data sources within relevant geographic units of analyses. Reported latitude and longitude coordinates were used to map prostitution charge events to corresponding census block groups (CBGs). We selected CBGs as the unit of analysis given that they are statistical divisions of census tracts and provide a granular unit by which to understand our data sources. The location of all prostitution charge data was then overlaid with other primary and secondary data to garner an overall sense of the distribution of sex work activities throughout Baltimore. We then calculated the number of prostitution charges per CBG and conducted windshield tours in those CBGs having at least the median number of charges (*n* = 3); in total, this generated a preliminary list of 42 CBGs for windshield tours. These CBGs ranged in size from 0.02 to 0.94 mile^2^ (median 0.10 mile^2^).

### Windshield Tour 1: CBG Level

As a way of verifying the locations of potential sex work identified through primary and secondary data sources, we conducted windshield tours (see Fig. 1; “Windshield Tour 1: CBG Level”) in the identified CBGs to confirm the presence of sex work activity. To ensure that study staff sufficiently covered the CBG, a GPS tracking smartphone application was employed to track staff routes. The tracking application was used as a quality assurance check to ensure that staff were covering all roadways in each CBG. Staff members also dropped pin drops if they saw a potential sex worker during the windshield tour. Although collecting pin drops of potential sex workers did not necessarily indicate confirmed sex workers, they did provide another layer of data for our TS strategy and reflect the ongoing investigative nature of the process.

The results of our first round of windshield tours at the CBG level did not yield significant insights into the specific locations street-based FSWs congregate. Many of the streets in the CBGs were residential in nature rather than true zones of sex work activity (i.e., it was evident that many CBGs had sex work strolls that were surrounded by larger residential areas) and resulted in long tours. Given staffing and fiscal limitations, and consideration of the overall geographic size of the CBGs, we concluded that we needed to revise our TS strategy such that we conducted cohort recruitment in much more precise locations with high concentrations of sex work activity.

#### Post Hoc Geospatial Analyses: City Block Level

In order to identify more precise locations of concentrated sex work activity, we refined our geospatial approach in three ways. First, we changed the geographical unit of analysis from the CBG level to the city block level, thus affording a much more granular lens to examine indicators of sex work activity. Second, rather than using the overall number of prostitution charges (triangulated with other data sources) in a given unit of analysis to inform our windshield tours, we conducted hot spot analyses at the city block level to identify clusters of sex work activity. Third, and related to our use of hot spot analyses, we conducted a series of hot spot analyses of prostitution charge data stratified by race and gender to account for the potential segregation of sex work activity (discussed below). Collectively, these refinements enabled us to derive a better understanding of the spatial distribution and clustering of sex work activity throughout Baltimore City and informed a second round of windshield tours at the city block level (see Fig. 1; “Windshield Tour 2: City Block Level”).

In accordance with the tenets of TS, our refined geospatial approach incorporated updated primary and secondary data, when applicable. We used the optimized hot spot analysis tool in ArcMap to identify spatial clusters of prostitution charge events from CY 2014. The optimized hot spot analysis tool “automatically aggregates incident data, identifies an appropriate scale of analysis, and corrects for both multiple testing and spatial dependence.” [25] This tool determines the settings that will “produce optimal hot spot analysis results.” [25] Because sex work activity may be fluid in nature, we set statistical significance at *p* < .10. Relaxed statistical significance allowed for the identification of a greater number of hot spots and better accounted for the practical significance of areas where FSWs may locate potential clients, such as strolls that span neighboring geographical divisions.

**Fig. 2:**
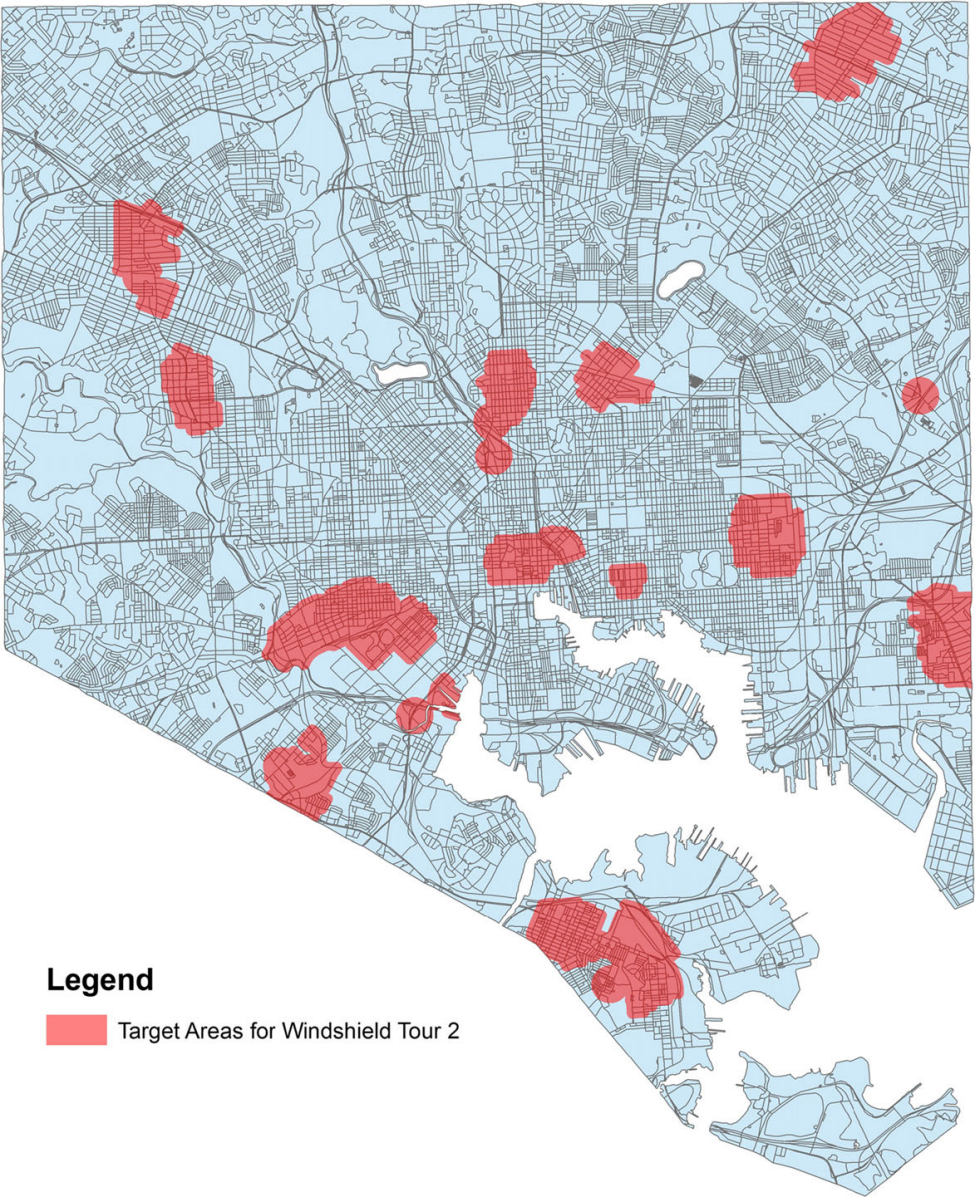
Target areas for Windshield Tour 2

As mentioned above, we conducted optimized hot spot analyses of the prostitution charge data stratified by race, limited to “black” and “white” given the small numbers of other race/ethnicities. Analyzing data by demographic groups allowed for a more nuanced understanding of potential racial segregation of FSW activities. Notably, we also added another layer to our hot spot analyses to include male-identified individuals charged with prostitution due to the potential overlap between areas where men and women sell sex. Due to small sample sizes, we did not analyze male prostitution charges by race. In total, we conducted five optimized hot spot analyses that reflected hot spots of the following: (1) all female prostitution charges; (2) all male prostitution charges; (3) all black female prostitution charges; (4) all white female prostitution charges; and (5) and all prostitution charges (across all genders). Following the creation of these hot spot maps, we merged the hot spots into a single layer in ArcMAP. Given our interest in recruiting street-based FSWs, we further refined our approach by applying a 300-ft buffer to the borders of the identified hot spots. Although applying a 300-ft buffer to the hot spots expanded the areas where we would do windshield tours, we felt it better accounted for the fluid nature of sex work. Next, we incorporated updated data to the city block level hot spots to further refine our windshield tours (see Fig. 1; “Windshield Tour 2: City Block Level”). First, we mapped updated ethnographic data of sex work activity. Because many of the locations from these data were non-specific (e.g., a stakeholder described a neighborhood or public park) and that street-based FSWs are mobile, we applied a 1000-ft buffer to their approximate point locations. Second, we incorporated updated data derived from postings made in the past month on the Johns website. Collectively, these refinements led to the identification of those areas shown in Fig. 2.

### Windshield Tour 2: City Block Level

Like the CBG level windshield tours, staff found the second round of windshield tours at the city block level to be long in duration. However, they did provide a more targeted canvassing of areas with high concentrations of sex work activity and allowed for continued pin dropping of potential sex workers. While the city block level windshield tours allowed us to verify locations of sex work activity, additional work was needed to understand location-specific contexts of sex work (e.g., exact times when sex work activity takes place, police presence, drug activities) and practical issues associated with field staff recruiting women in these environments, such as safety concerns and van parking. As a result, we elected to conduct additional geospatial analyses (discussed below) and implement walking tours. During the walking tours, we conducted key informant interviews with constituents to access their knowledge of sex work activity as well as glean information about safe locations for staff to recruit participants and park the study van.

### Walking Tours

To identify where we should conduct the walking tours, we created heat maps of 911 call center data and sex worker pin drops that were collected during the police ride-alongs and windshield tours (see Fig. 1; “Walking Tour”). Heats maps were used as they allow for examinations of the relative density of point features, such as prostitution reports. We elected to use these two data sources as the foundation for guiding where we conducted walking tours as they may have reflected a more realistic measure of sex work activity than prostitution charge data given that charge data is reflective of police capacity to conduct stings. Furthermore, we knew that prostitution reports would drive where police conducted prostitution stings and therefore overlap with where charges occurred. Collectively, this approach enabled us to build upon what we learned from the windshield tours by focusing our walking tours in highly precise locations that were primarily within the CBGs and city blocks previously toured.

Due to the 911 data limitations (i.e., data did not include information pertaining to how many unique persons reported sex work activity), we chose to create two heat maps to inform our walking tours: one using only 911 call center data and another using both 911 call center data and sex worker pin drops. This approach was also used because we did not have enough pin drops to conduct meaningful heat mapping using *only* those data. Through these maps, we were able to increase the specificity of our understanding of where sex work activity took place. For example, the heat map that used only 911 call center data suggested that there were small geographic areas with high concentrations of sex work activity. Building on this map, incorporation of the sex worker pin drops did not change the relative locations of areas with greatest concentrations of sex work activity, but rather expanded their geographic footprint, thus enhancing how we understood the fluidity of sex work.

We found that heat maps offered significant utility in enhancing our understanding of sex work activity by reflecting the highest concentrations of sex work activity. The identified locations for the walking tours encompassed a large portion of the 911 report data. Specifically, from March 2015 to December 2015, there were 2193 prostitution reports made to the 911 call center. Of these, 98.4% (*n* = 2159) included data viable for geocoding. In comparing the locations of the walking tours to the locations of the 911 reports, 63.7% (*n* = 1375) of the 911 reports were within the areas where we conducted walking tours. Further, 83.5% of the pin drops of potential sex worker identified during the first and second windshield tours were within the target areas. During the walking tours, we engaged key informants (*n* = 90) about sex work activities and found that although their accounts corroborated that sex workers were in the area, they were not able to describe in great detail when sex workers were out.

### Development of the Sampling Frame

After completing the walking tours, we operationalized the units of our TS frame (see Fig. 1; “Sampling Frame”). Comparable to time location sampling (TLS) [26], each unit of our sampling frame consisted of three components: a venue, day of the week, and specific time period within a day. While the development of our TS frame parallels those methods used in TLS, they are distinct in that we focused our recruitment efforts in those locations with highest concentrations of sex work activity rather than a random selection of the “universe” of areas of sex work activity (as would be done in TLS). The location (venue) components of our sampling frame units were defined as a small geographical area with high concentrations of sex work activity, collectively identified via information derived from our geospatial analyses of primary and secondary data sources and resulting windshield and walking tours. In total, we identified 14 venues with high concentrations of sex work activity for participant recruitment in Baltimore City.

The day and time components of the sampling frame were guided by qualitative data collected during the walking tours; however, because these data did not provide a great deal of insight into specific times of sex work activity, we supplemented these data with information derived from the 911 call center data. Specifically, we calculated overall frequency distributions of sex work reports to the 911 call center by day and time. We then selected those days and times in each location that suggested high concentrations of FSW activities. Furthermore, day and time units were also triangulated with primary and secondary data sources. Given the transient nature of street-based sex work, we elected to group the time component of the sampling frame in 4-h blocks. This ensured maximum coverage during peak hours of potential sex work activity in each venue. In total, our 14 locations generated 71 unique venue-day-time units for the sampling frame.

Importantly, we did not set recruitment targets for the units of our TS frame due to the absence of FSW population size estimates in Baltimore City. Furthermore, the charge data did not include information that would have allowed us to ascertain the number of unique persons charged with prostitution, nor did the 911 call center data provide a unique identifier for the person that made the report. Similar to research [27] among opioid-dependent persons in Baltimore, the presence of sex work activity throughout many different parts of Baltimore City also impeded our ability to set quotas for each venue.

## Results

**Fig. 3:**
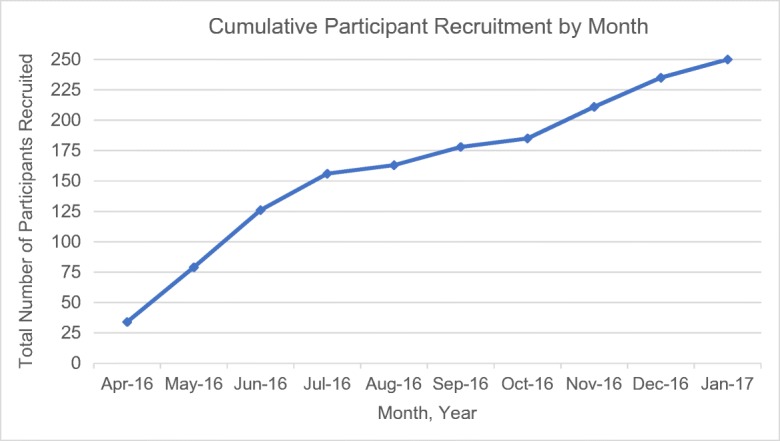
Cumulative participant recruitment by month

Our baseline recruitment activities spanned April 2016 to January 2017 (Fig. 3). Because we anticipated challenges in recruiting FSW partly due to negative interactions with police and other community stakeholders, we chose to visit all of the units of our sampling frame. Study staff visited randomly selected units of the sampling frame with FSW activities on the corresponding day in which recruitment activities were taking place; for example, if staff were conducting recruitment on a Tuesday, staff selected randomly from those units of the sampling frame that indicated FSW activities occurred on Tuesdays. After visiting all of the units of the sampling frame, we re-evaluated our data sources and developed a supplementary list of sampling frame units (*n* = 13); notably, these units largely paralleled our initial list of 71-day time units, but with marginally different time components. In total, we recruited a cohort of 250 FSWs over 129 shifts, totaling 555 h in which our recruitment van and associated field staff were in the identified areas of sex work activity. On average, each participant took 2.2 h to recruit in the field with approximately two participants recruited per shift. As an incentive for participating in this research, all FSWs received a pre-paid $70 USD VISA gift card for completing the baseline survey. Although the iterative nature of our TS strategy was analytically labor intensive and required windshield and walking tours, it maximized efficiency in cohort recruitment via its specificity and ability to target the precise times and locations of peak sex work activity.

## Discussion

This research describes a robust targeted sampling approach used to recruit one of the first cohorts of street-based FSWs in the USA. Our experiences demonstrate that while the ongoing investigative nature of TS may be analytically labor intensive, it can be successfully utilized in a modern era of diverse secondary data sources to efficiently recruit a sample of street-based FSWs in the USA. Unique data sources paired with ethnographic data and ongoing data collection on the target population during windshield and walking tours allowed for the development of a comprehensive and up to date understanding of the geotemporal distribution of street-based sex work activity in Baltimore City. This research builds on existing studies that employed TS by showing how researchers may employ the method in other places where relevant data sources are publicly available online and constantly updated. Our study also enhances the TS literature by demonstrating how multiple layers of geospatial analyses can be used to create a sampling frame for recruiting a diverse cohort of study participants. The inherent flexibility of TS afforded our study the ability to adapt to the respective limitations of our data sources in real time, while rigorously evaluating where to best implement recruitment efforts.

Numerous lessons were learned throughout the implementation of our TS strategy. First, the employment of a diversity of secondary data sources adds to the comprehensiveness and robustness of identified recruitment venues. Our use of data derived from a Johns website led to the identification of areas of sex work activity that were not identified via prostitution charge data and 911 call center data. Another lesson learned was that of the importance of conducting multiple layers of geospatial analyses to identify areas of sex work activity. By analyzing available data by specific demographics, we identified areas of sex work activity that were not captured by macro-level analyses. Relatedly, by relaxing statistical significance to *p* < .10 for hot spot identification and applying buffers to identified venues, we were able to better ensure our research team conducted tours in all potential areas of sex work activity. Collectively, these steps in our TS strategy afforded us the ability to target precise times and locations where street-based FSWs congregate while still accounting for persons’ relative mobility. This was a significant benefit to our study as street-based FSWs are both a hidden population and subject to external factors, such as police presence, that may influence when and where sex work takes place.

We also learned that windshield tours can be burdensome on staff if persons are not given precise locations to tour. For example, in our CBG-level windshield tours, the target areas were simply too large. While windshield tours may offer considerable insights into the behaviors of the target population, researchers must be cognizant of the burden created by canvassing these areas and assess overall feasibility of touring an identified area in entirety. Relatedly, researchers should evaluate if the areas they identify for windshield tours are of true interest or rather larger areas that contain small, concentrated areas of the target population. For example, we quickly learned during our CBG-level windshield tours that the CBGs likely contained FSW strolls that were surrounded by larger residential areas in which sex work did not occur. Furthermore, researchers may be well served to include quality assurance checks; our use of a GPS tracking application served as an effective strategy to verify where staff were going during the windshield tours.

There were multiple strengths of this research. First, the complexity and diversity of data sources afforded our team a robust understanding of sex work activity in Baltimore City. Furthermore, our geospatial analyses and associated tours ensured that data were evaluated at the appropriate unit of analysis while still allowing for ongoing data triangulation and collection (e.g., pin drops during tours). Relatedly, our inclusion of hot spot analyses specific to each demographic group paired with ethnographic data and information gleaned from the Johns website enhanced our understanding of sex work throughout Baltimore City.

This research is not without limitations. There was a degree of circularity between the 911 call center data and prostitution charge data as policing efforts, and therefore, arrests are responsive to community demands. Relatedly, the police data only included the top charge. As a result, it is possible FSWs who were charged with higher-level crimes at the time of receiving a prostitution charge were not reflected in our analyses. Another limitation of this research is that we only engaged FSWs directly in understanding the locations of their sex work activities via our community advisory board. Conducting ethnographic data collection with a larger and more diverse group of FSWs may have led to increased understanding of sex work activity; however, this population is difficult to engage in research without first developing a preliminary understanding of where and when sex work occurs and cultivating relationships with community gate keepers. Lastly, while our use of hot spot and heat mapping resulted in the identification of areas with high concentrations of sex work activity, it is possible we did not access FSWs that work in more isolated areas. In summary, we feel the efficiently with which we recruited our cohort, and cohort diversity, outweigh these limitations.

## Conclusion

TS is a productive strategy to recruit members of hidden populations that affords flexibility in participant recruitment via incorporation of new data that describes when and where the target population may be found. Purposeful geospatial analyses of diverse and emerging forms of publicly accessible data paired with traditional forms of data can lead to the rapid identification of where and when members of the target population congregate. Although the iterative nature of TS may be analytically labor intensive, it can be a productive strategy for the recruitment of hidden populations.
